# Editorial: Mechanisms and Strategies of Arthropod Adaptation to the Chemical Environment

**DOI:** 10.3389/fphys.2022.889757

**Published:** 2022-03-25

**Authors:** Chaoyang Zhao, Fang Zhu, Qian Sun, Xuguo Zhou

**Affiliations:** ^1^ Center for Medical, Agricultural and Veterinary Entomology, United States Department of Agriculture, Agricultural Research Service, Gainesville, FL, United States; ^2^ Department of Entomology, Pennsylvania State University, University Park, PA, United States; ^3^ Department of Entomology, Louisiana State University, Baton Rouge, LA, United States; ^4^ Department of Entomology, University of Kentucky, Lexington, KY, United States

**Keywords:** arthropod, adaptation, sequestration, pesticide, RNAi

As one of the most successful groups of animals, arthropods have evolved a wide range of adaptive strategies that allow them to live in almost every habitat on Earth (Ledesma et al., 2020). These strategies largely involve in the capabilities of coping with the chemical stresses imposed by their environments, including both biotic and abiotic components, to help them survive and thrive (Korsloot et al., 2004). While the biotic components can be the hosts, predators, parasitoids, and competitors of arthropods, pesticides have become an increasingly prominent abiotic factor owing to their extensive/indispensable use in agricultural and urban environment (Sparks and Nauen, 2015; Gould et al., 2018). Nevertheless, both biotic and abiotic components have been some of the key drivers facilitating the evolution of stress management in arthropods, which include perceiving, processing, and responding to chemical signals at a variety of biological levels (Després et al., 2007; Vilcinskas, 2013; Liu, 2015; van Leeuwen and Dermauw, 2016; Alyokhin and Chen, 2017). This Research Topic is dedicated to this topic and the following four papers have advanced our understanding by examining pertinent hypotheses.

To defend against predators, certain herbivorous arthropods evolve the ability of utilizing toxic chemical compounds produced by their host plants as molecular weapons, a research field that has drawn growing attention in recent years (Petschenka and Agrawal, 2016). Instead of metabolizing them, some arthropods can absorb and accumulate these plant compounds in their body, thereby making themselves toxic or unpalatable to their predators, a phenomenon termed sequestration (Nishida, 2002; Beran and Petschenka, 2022). As one of the best-known examples, the horseradish flea beetle, *Phyllotreta armoraciae*, a monophagous insect feeding on brassicaceous plants, is able to sequester host-derived glucosinolates to protect itself against predators (Yang et al., 2020). In this Research Topic, Yang et al. further showed that the uptake of glucosinolates mainly occurred at the foregut of *P. armoraciae*, in contrast to the widely accepted notion that the endodermal midgut is the tissue for hydrophilic compound absorption. According to authors, and as far as we are aware, this is the first report that insects may use their foregut to absorb hydrophilic compounds, laying the ground for understanding the roles foregut may play in insects’ adaptation to the chemical environment.

The metabolism of toxic compounds is another way that arthropods use to survive the natural and synthetic chemicals (Li et al., 2007). Unlike sequestration whose research is still in its infancy, xenobiotic metabolism has been extensively studied, and multiple classes of detoxification enzymes have been identified and functionally characterized, including cytochrome P450 monooxygenases (P450s), glutathione S-transferases (GSTs), carboxylesterases (CarEs), UDP-glucosyltransferases (UGTs), sulfotransferases, and ATP-binding cassette (ABC) transporters (Feyereisen 2012; Zhu et al., 2014; van Leeuwen and Dermauw, 2016; Nauen et al., 2022). Two papers in this issue investigated the mechanisms underlying pesticide detoxifications using the insect pests of public health importance, the housefly, *Musca domestica*, and the southern house mosquito, *Culex quinquefasciatus*, respectively. While Gong et al. focused on the role of cytochrome P450 reductase (CPR) as a cofactor of P450s in pesticide metabolism, You et al. examined the diel rhythmic expression of several detoxification genes, including those encoding P450s, GSTs, and CarEs, and discussed how such expression scheme was associated with pesticide susceptibility in these insects.

RNA interference (RNAi) is a gene silencing mechanism that arthropods, as many other life forms, have evolved to circumvent viral infection (Fire et al., 1998; Wilson and Doudna, 2013). This mechanism has been used to develop the strategies for beneficial arthropod protection and pest control (Vogel et al., 2019). The Bayer “SmartStax Pro” maize (Mon87411), the first RNAi transgenic trait, has been recently deregulated in the US, China, and Canada, and this RNAi-based biocontrol product is commercially available to the US farmers, starting 2022 (De Schutter et al., 2022). By allowing the target arthropod pests to ingest double-stranded RNA (dsRNA) molecules that function to silence specific genes, pests are killed or their viability is impaired. However, the efficacy of RNAi can be affected by many factors including the instability of dsRNAs prior to their entry into host cells. The fourth paper by Lei et al. identified and characterized the sole *dsRNase* gene in the tawny crazy ant, *Nylanderia fulva*, to improve the silencing efficacy for this emerging invasive pest that spreads rapidly across the southern United States.

Lastly but certainly not least, we are grateful to all authors for contributing their articles and anonymous reviewers, as well as editorial staff for their constructive comments and suggestions. We hope this Research Topic will be of interest to the broad readership of Frontiers in Physiology.
